# Cancrum Oris

**Published:** 1891-04

**Authors:** W. P. Fleming

**Affiliations:** Georgetown


For Daniel's Texas Medical Journal.

### CANCRUM ORIS.

BY W. P. FLEMING, M. D., GEORGETOWN.

Read before the West Texas Medical Association.

ANY INFLAMMATION of the mouth may be attended with ulceration, but the complaint here referred to is essentially ulcerative, appearing in this form at the commencement, and presenting characters which entitle it to rank as a distinct affection. The complaint usually makes its appearance in the gums or inside of the cheeks or lips, though it may occur in any part of the mouth, or in the fauces. When first noticed it is in the form of an ulcer, often of considerable size, with a yellowish-white or grayish surface, and an inflamed border. The neighboring parts are also inflamed and swollen, and, when the ulcer is in the cheek or lips, the tumefaction is observed externally, the cheek of the side affected being red, shining and prominent. The swelling in the mouth is sometimes so considerable as to render an examination of the sore difficult. There is a very copious flow of saliva, and the breath is very offensive, though the fetor is distinct from that of gangrene.

The causes of cancrum oris are obscure. It generally occurs in children from two to six years old, has been ascribed to a debilitated habit of body, arising from deficient or improper food, bad air, etc., and I think in a great measure to malarial influences.

I was called to see Mrs. C., aged 23 years, who had been subject during the fall to spells of intermittent fever, for which antiperiodics had been given, disposing of the fever, which fevers originated from exposure in a tent to which she was unused, during the summer and fall months, and the prevailing malarial cause of the locality. On the 15th of March, at the close of the last intermittent, some swelling of the upper portion of the face occurred, and about the eyes, on the two succeeding days swelling or the throat and cheeks took place, during which time the peculiar smell of cancrum oris was perceptible. Upon examination the fauces and left cheek were found inflamed, as well as the tongue to some extent. Situated upon the soft palate, immediately on the right side of the uvula, was situated a buckskin-colored patch of dead tissue, eliptic in shape, about the size of a lima bean.

Intermittent returned on the 23d, for which she was prescribed for on the 21st; a slight intermittent on the 21st; which went off without subsequent return. During the 20th much more nausea than usual was experienced, strong inference arose that it proceded from the swelling of the dead tissues, for at that time upon examination it was found to be cast off from the surface of which it had formed a part, leaving some remnants of it still adhering. On the 23d the surface was free, granulating and healthy, without fever, and without difficulty and inconvenience in swallowing or eating. My treatment in this case was a tonic for the beginning, of tinc. ferri mur. and quinine, touched ulcers with a solution of sulph. zinc. 15 gr. to the ounce of water; this was applied four times a day.

